# Forward Genetics by Genome Sequencing Uncovers the Central Role of the *Aspergillus niger goxB* Locus in Hydrogen Peroxide Induced Glucose Oxidase Expression

**DOI:** 10.3389/fmicb.2018.02269

**Published:** 2018-09-24

**Authors:** Thanaporn Laothanachareon, Juan Antonio Tamayo-Ramos, Bart Nijsse, Peter J. Schaap

**Affiliations:** ^1^Laboratory of Systems and Synthetic Biology, Wageningen University and Research, Wageningen, Netherlands; ^2^Enzyme Technology Laboratory, National Center for Genetic Engineering and Biotechnology, Pathumthani, Thailand; ^3^International Research Centre in Critical Raw Materials-ICCRAM, University of Burgos, Burgos, Spain

**Keywords:** forward genetics, genome sequencing, *Aspergillus niger*, N402, *goxB*, glucose oxidase

## Abstract

*Aspergillus niger* is an industrially important source for gluconic acid and glucose oxidase (GOx), a secreted commercially important flavoprotein which catalyses the oxidation of β-D-glucose by molecular oxygen to D-glucolactone and hydrogen peroxide. Expression of *goxC*, the GOx encoding gene and the concomitant two step conversion of glucose to gluconic acid requires oxygen and the presence of significant amounts of glucose in the medium and is optimally induced at pH 5.5. The molecular mechanisms underlying regulation of *goxC* expression are, however, still enigmatic. Genetic studies aimed at understanding GOx induction have indicated the involvement of at least seven complementation groups, for none of which the molecular basis has been resolved. In this study, a mapping-by-sequencing forward genetics approach was used to uncover the molecular role of the *goxB* locus in *goxC* expression. Using the Illumina and PacBio sequencing platforms a hybrid high quality draft genome assembly of laboratory strain N402 was obtained and used as a reference for mapping of genomic reads obtained from the derivative NW103:*goxB* mutant strain. The *goxB* locus encodes a thioredoxin reductase. A deletion of the encoding gene in the N402 parent strain led to a high constitutive expression level of the GOx and the lactonase encoding genes required for the two-step conversion of glucose in gluconic acid and of the *catR* gene encoding catalase R. This high constitutive level of expression was observed to be irrespective of the carbon source and oxidative stress applied. A model clarifying the role of GoxB in the regulation of the expression of *goxC* involving hydrogen peroxide as second messenger is presented.

## Introduction

*Aspergillus niger* is a highperformance microbial cell factory and used for cost effective production of food additives, pharmaceuticals and industrial enzymes. Having the abilities of a natural tolerance toward acid media as well as a high secretory capacity, *A. niger* is exploited as a cell factory to produce citric and gluconic acid (Meyer et al., 2011). Being saprobic, it is additionally equipped with versatile catabolic properties that make it grow on many kinds of, often complex, organic waste materials under various environmental conditions.

*A. niger* strain N402 (ATCC 64974) is a laboratory work horse strain that has been extensively used as a master strain for the generation of regulatory and structural mutants, with the aim to provide genetic insight in the molecular mechanisms underlying its extraordinary abilities. Additionally, the strain has been frequently used as model cell factory for overproduction of endogenous and heterologous enzymes (Lubertozzi and Keasling, 2009), to improve production of green chemical building blocks such as succinic acid, malic acid (Meyer et al., 2011), and itaconic acid (van der Straat et al., 2014), and as a model system to study the roles and regulation of individual sugar transporters in the catabolism of plant material feedstock components (Sloothaak et al., 2014, 2015, 2016a,b). Laboratory strain N402 is a derivative of the wild type strain N400 (CBS 120.49, NRRL3, ATCC 9029) and was obtained after two successive rounds of low-dosed UV mutagenesis (Bos et al., 1988; Swart et al., 2001). As a result, N402 showed a phenotype different from its origin, notably the development of shorter conidiophores (*csp*A1), a property that makes this strain much more amendable to genetic manipulations in a laboratory setting than wild type strains.

Organic acid production by *Aspergillus* species is tightly linked to biotechnological production of commodity chemicals. *A. niger* strain N400 was originally characterized as an efficient gluconic acid producer (Blom et al., 1952), a multifunctional carbonic acid that is used as a bulk chemical in the food, pharmaceutical, and cement industries (Singh and Kumar, 2007; Yang et al., 2017). Gluconate formation by *A. niger* occurs extracellularly and therefore does not require the involvement of intracellular metabolic pathways. Nevertheless, accumulated genetic evidence (Swart et al., 1990; Witteveen et al., 1992) suggested that the regulation of GOx expression is a complex trait implicating at least seven complementation groups and that hydrogen peroxide, a product of the first step of GOx mediated biotransformation of glucose to gluconic acid, acts as a possible signaling metabolite for GOx induction (Witteveen et al., 1993). As the underlying molecular mechanisms are still not understood we aimed to apply forward genetics as an approach to identify genes responsible for this trait.

Unraveling a polygenic trait by forward genetics requires the generation of a correspondingly large set of mutants, a well-defined genetic background, and laborious complementation tests to group and genetically map them to a locus. A mapping-by-sequencing method can then be applied to identify the causal genes for further study (Schneeberger, 2014). While for the GOx system the genetic requirements have been met in the past (Swart et al., 1990; Witteveen et al., 1992, 1993) and even though, strain N402 is universally applied in *Aspergillus* research, its genome information has not yet been published. In this study, a high-quality draft of the N402 genome sequence was obtained by using a hybrid of Illumina and PacBio RS II sequencing techniques. Phenotypic analysis of GOx overproducing mutants indicated a pivotal role of the *goxB* locus as strains NW102:*goxB12* and NW103:*goxB21* showed GOx expression virtually independent of the carbon source, at a low pH (Swart et al., 1990) and under conditions of low oxygen availability (Witteveen et al., 1993). Using a mapping-by-sequencing approach the gene linked to the *goxB* locus, was identified.

The effect of a subsequent deletion in the parental strain of the *goxB* gene, encoding a thioredoxin reductase, was further evaluated by plate assay and transcriptional analysis using different oxidative stresses and carbon sources. Deletion of the thioredoxin reductase encoding gene led to the *goxB* phenotype: a high constitutive expression of the genes coding for GOx, lactonase A (EC 3.1.1.17), and catalase R (EC 1.11.1.6). A model clarifying the role of the *goxB* locus in the regulation of GOx expression, involving hydrogen peroxide as second messenger, is presented.

## Materials and Methods

### Strains and Growth Conditions

*A. niger* N402 (*cspA*1) (Bos et al., 1988) has been used as master strain for the acquisition of *gox* mutant strains. *A. niger* NW102 (*goxB*12, *cspA*1, *pabA*1) and NW103 (*goxB*21, *cspA*1, *pabA*1) harbor the *goxB* mutations. *A. niger* strain MA169.4 (*kusA*::DR-*amdS*-DR, *pyrG*^−^) is descendant of N402 and was used as recipient strain in the knock-out transformation experiment (Carvalho et al., 2010) using the *pyrG* gene from *A. oryzae* as a selectable marker gene. The pWay-pyrA plasmid was used as a control for transformation. *A. niger* strains were maintained on complete medium containing 1 g/L casamino acid, 5 g/L yeast extract, 1% glucose, 20 mL/L ASPA + N, 1 mL/L Vishniac solution, and 1 mM MgSO_4_ at 30°C (Arentshorst et al., 2012).

### Isolation of Genomic DNA of *A. niger* for Whole Genome Sequencing

Mycelial biomass of strains N402 and NW103 was obtained by growth in 100 mL complete medium at 30°C and 200 rpm for 40 h, washed with sterile demi water and dried. The dried mycelium was snap frozen in liquid nitrogen and grinded in liquid nitrogen into a fine powder with a pre-chilled mortar. The powder mycelium was re-suspended in 0.8 mL DNA extraction buffer (0.1 M Tris-HCl, pH 8.0, 1.2 M NaCl, and 5 mM EDTA) and mixed by vortexing. Next, 15 μL of RNase A (10 mg/mL) was added and the solution was incubated at 65°C for 30 min. followed by phenol:chloroform:isoamylacohol (25:24:1) extraction and isopropanol precipitation. Genomic DNA was re-suspended in 50 μL of milliQ water. The concentration was determined using the Qubit^®^ RNA HS Assay Kit (Thermo Fisher Scientific, MA, United States).

### Genome Sequencing, Assembly, Annotation, and Bioinformatics

#### Sequencing

Whole genome sequencing of strain N402 was performed by a commercial vendor (Novogene Bioinformatics Technology Co. Ltd, Beijing, China.) using Illumina HiSeq (300 bp inserts library with 150 bp paired-end sequencing; Illumina, San Diego, CA, United States) and PacBio RS II platform (10 kb inserts library; Pacific Biosciences, Menlo Park, CA, United States).

#### Assembly

A *de novo* IDBA_UD (Peng et al., 2012) based assembly pipeline A5 v20160825 (Coil et al., 2015) was used to assemble the HiSeq reads into contigs. Scaffolding on the contigs was performed with the AHA Long Read v1 protocol in the PacBio SMRT Analysis v2.3.0.140936 software suite. The following parameter setting were used: Normal Coverage, Gapfill true, 6 iterations. This was followed by another scaffolding round using SSPACE Longread v1-1 (Boetzer and Pirovano, 2014) with a Maximum link ratio of 0.1. MEDUSA v1.6 (Bosi et al., 2015) for reference scaffolding with *A. niger* ATCC 1015 strain as reference. Bowtie2 v2.3.0 (Langmead and Salzberg, 2012), samtools v1.3.1 (Li et al., 2009), and Pilon v1.21 (Walker et al., 2014) with the HiSeq reads for automatic assembly improvement. Finally, low coverage (<10) and small size contigs (<500 bp) were removed. HiSeq and PacBio Reads were mapped with bowtie2 v2.3.0 on the scaffolds for manual inspection with Tablet v1.16.09.06 (Milne et al., 2013), breaking the scaffolds at locations with low quality coverage. QUAST v4.4 (Gurevich et al., 2013) was used or assembly quality assessment.

#### Annotation

For genome annotation, the N402 assembly was imported in SAPP semantic database (Koehorst et al., 2017). STAR v.2.5.2b (Dobin et al., 2013) was used for mapping RNAseq data from Odoni et al. (2017) on the assembly and BRAKER1 (Hoff et al., 2016) for unsupervised RNA-Seq-based gene prediction. Gene predictions were directly stored in the SAPP semantic database (Koehorst et al., 2017). Structural feature description was done using the GBOL ontology (van Dam et al., 2017) Functional genome annotation was done with a standalone version of interproscan v5.24.63.0 (Zdobnov and Apweiler, 2001) in direct interaction with the SAPP database using the pfam31 (Bateman et al., 2004) database. The raw reads and full genome sequence are available from ENA (accession numbers PRJEB21769 and GCA_900248155).

#### Variant Calling

bowtie2 v2.3.0, samtools v1.3.1, VarScan2 v2.3.9, (Koboldt et al., 2012) and Tablet v1.16.09.06 for manual inspection of SNPs between N402 and NW103.

SignalP4.1 (Petersen et al., 2011) was used to detect the signal peptide for secretion in the putative lactonase amino acid sequences. Clustal Omega (Sievers et al., 2011) was used for multiple sequence alignment of putative lactonases and fungal TrxR strains. The structure of the yeast thioredoxin reductase A chain was visualized using Cn3D (Wang et al., 2000).

### Genetic Construction of the *goxB/trxR* Knock-Out Strains

Using the split-marker approach, the *goxB*/*trxR* gene was deleted from the genome of the MA169.4 strain (isogenic of N402), which is defective in the Non-Homologous End-Joining (NHEJ) pathway through a transiently silenced *kusA* gene (Arentshorst et al., 2015). A schematic representation of the experimental steps required can be found in **Supplementary Figure S1**. Correct deletion of the gene was confirmed with PCR. Primers used are listed in **Supplementary Table S1**.

### Protoplast Preparation and Transformation

A protoplast-mediated transformation of *A. niger* was performed by adapting the protocol of Arentshorst et al. (2012). The spore at 1 × 10^6^ spores/mL medium was inoculated in 250 mL complete medium with 10 mM uridine in a 1 L erlenmeyer flask coated with 5% dimethydichlorosilane in heptane. The culture was incubated at 30°C and 100 rpm for 12 h. The mycelium was harvested by filtration through sterile miracloth and washed once with SMC buffer. Novozyme234 was used for protoplast formation (100 mg Novozyme234 per g of mycelium). Mycelium was incubated at 37°C and 80 rpm for 3 h. Protoplasts and mycelium were separated by filtration through the miracloth. The filtrate containing the protoplast was centrifuged at 2,000 g and 10°C for 10 min washed twice with 1 mL STC buffer and re-suspended in 1 mL STC buffer and aliquoted to 100 μL per reaction. After transformation, single *A*. *niger* transformant colonies were purified and the transiently silenced *kusA* gene was restored on MM plates containing fluoroacetamide (FAA) as described (Carvalho et al., 2010).

### Glucose Oxidase Agar Plate Assay

Colonies were stained for glucose oxidase (GOx) using a modified method described by Witteveen et al. (1990). The solid assay medium contained 6 g/L, NaNO_3_, 1.5 g/L KH_2_PO_4_, 0.5 g/L KCl, 0.5 g/L MgSO_4_.7H_2_O, 1 mL/L Vishniac trace element solution, 12 g/L agar, and supplemented with 2.5 mM *o*-anisidine and one of the indicated carbon sources including 2 or 50 mM glucose, 50 mM fructose, 50 mM acetate, or 50 mM gluconate. The spores were diluted to 1 × 10^7^ spores/mL and then 5 μL of spore suspension solution was dropped onto the agar plate. The agar plates were incubated at 30°C for 40 h. After incubating, the staining solution containing 20 mM sodium phosphate buffer, pH 7.0, 0.1 mM glucose and 20 μg/mL horseradish peroxidase was added into the plates and then incubated at room temperature for 4 h.

### RNA Extraction and Transcriptional Analysis of the GOx System

For carbon source shift experiments, the wild type N402 and the Δ*trxR* strains were pre-cultured at 1 × 10^6^ spores/mL at 30°C and 200 rpm, during 18 h, in minimal medium containing 4.5 g/L NaNO_3_, 1.13 g/L KH_2_PO_4_, 0.38 g/L KCl, 0.38 g/L MgSO_4_.7H_2_O, 2 g/L casamino acid, 1 g/L yeast extract, 1 mL/L Vishniac trace element solution and supplemented with 100 mM sorbitol. Mycelium was harvested and then rinsed with equal amounts of water and transferred to minimal medium with either 100 mM sorbitol, 2 mM, and 50 mM glucose, 50 mM fructose, 50 mM acetate, or 50 mM gluconate. The initial pH of the medium in all condition was set at 6.0. After 4 h incubation, mycelium transfer samples were taken for RNA isolation, quickly washed, and then dried with a single-use towel, snap-frozen with liquid nitrogen and stored at –80°C until further processing. Two biological replicates per condition were studied in all cases.

Oxidative stress experiment was performed similar to the carbon sources experiment. The wild type N402 and the Δ*trxR* strains were pre-cultured in minimal medium supplemented with 50 mM fructose. The water-rinsed mycelium was transferred to minimal medium supplemented with 50 mM fructose with the following oxidizing agents: 75 mM H_2_O_2_, 1.8 mM diamide, 0.8 mM menadione, and incubated at 30°C for 3 h. Concentrations of the stress initiating agents and exposure times were taken from (Pócsi et al., 2005).

Transcriptional analysis of *A. niger* genes: RNA of *A. niger* mycelium was isolated as described in Sloothaak et al. (2016b). Reverse transcription, quantitative PCRs and calculation were performed following the protocols and instrument described in Mach-Aigner et al. (2012). The previously described histone H4-like transcript (ATCC64974_101030, ATCC 1015 gene ID 207921) was used as reference for normalization of the expression data. Primer sequences are in **Supplementary Table S1**. Cycling conditions and control reactions were done as described previously (Steiger et al., 2010).

## Results

### Genome Sequencing of Strain N402

**FIGURE 1:**
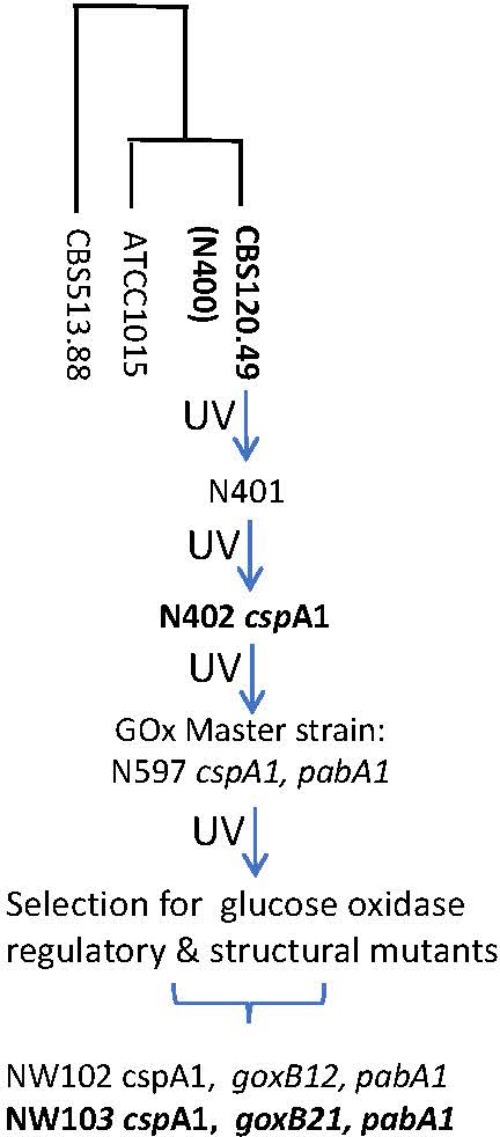
Pedigree of glucose oxidase regulatory and structural mutants of *A. niger* strain N400. For strains in bold genome sequence information was obtained. N400 derived genotypes are in italics; *pabA1*, *p*-aminobenzoic acid deficient. The “short conidiophores” *cspA1* genotype was obtained after two rounds of UV mutation and selection and therefore most likely represents a complex trait. The construction of the GOx master strains have been described by Bos et al. (1988). The loci of glucose oxidase regulatory and structural mutants have been described by Swart et al. (1990). The genomes of strains CBS513.88 (Pel et al., 2007) and ATCC1015 (Andersen et al., 2011) are used for reference purposes. UV, UV mutagenesis.

An overview of the bioinformatics workflow is depicted in **Figure 1**. The most recent common ancestor of the seven GOx complementation groups is strain N402. Since the N402 master strain was first selected for having the *cspA1* phenotype, which could only be obtained after 2 consecutive rounds of UV mutagenesis and selection, we surmised that this trait may also be polygenic, and that the N402 master strain could have acquired multiple unlinked mutations which are directly passed on to the NW102 and NW103 *goxB* strains. As this could hamper molecular identification of specific loci, we first obtained a high-quality *A. niger* N402 draft genome sequence using a hybrid of Illumina and PacBio RS II sequencing, and subsequently mapped NW103 Illumina reads against the scaffolds, while focusing on linkage group II. An overview of the sequencing and assembly strategy is provided in the method section. As detailed in **Table 1**, the combination of the Illumina (150 nt paired end reads and ∼200 times coverage) and PacBio RS II sequencing technologies (library insert length 10 kbp and ∼30 times coverage) yielded a high quality 35,5 Mb draft genome. Three of the eight nuclear chromosomes seemed to be complete, as scaffolds spanning their entire length were obtained (**Table 1**).

**Table 1:** Overview of the *A. niger* N402 genome draft.

Linkage group	Scaffold(s)	Scaffold size in bp	#Genes^∗^	Remarks
I	N402_101 N402_102	2,261,712 1,182,502	755372	
II	N402_200	4,812,097	1,535	Complete; contains the *goxB* and *goxC* locus
III	N402_301 N402_302 N402_303	2,069,061 1,922,870 1,232,819	657594406	
IV	N402_401 N402_402 N402_403	5,108,653 118,3368 132,900	1,58338744	
V	N402_501 N402_502 N402_503	2,719,173 693,317 3,009	83622914	
VI	N402_601 N402_602 N402_603	2,870,019 810,074 570,523	860271173	rDNA repeat
VII	N402_700	2,896,446	912	Complete chromosome
VIII	N402_800	5,004,651	1,594	Complete chromosome
	N402_Scf116 N402_Scf129	43,824 17,555	104	
Total	19	35,561,656	11,236	

*^∗^As obtained by Braker1 RNAseq guided ab initio gene prediction.*

**FIGURE 2:**
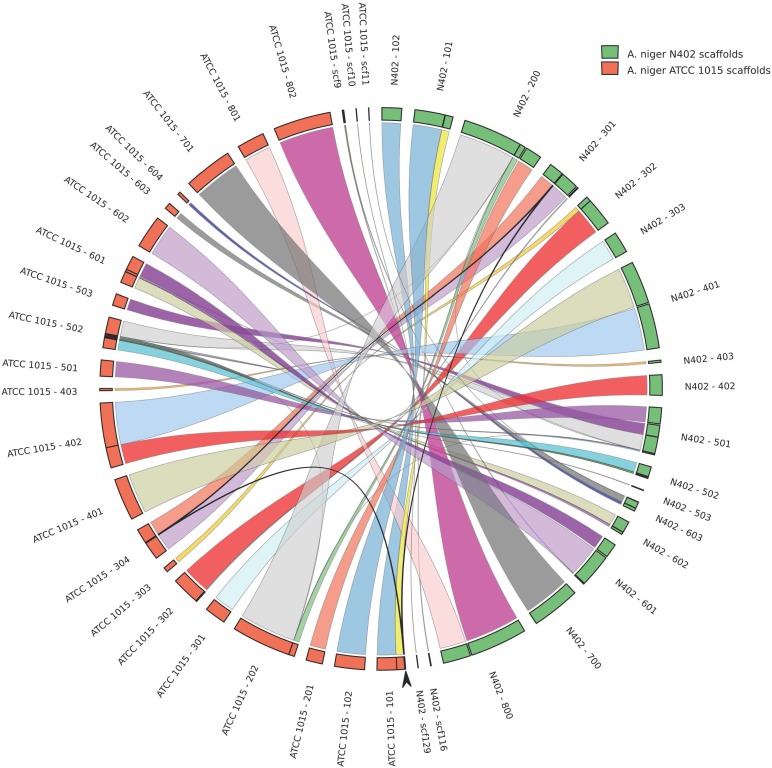
High levels of sequence identity and synteny between the proposed genome assemblies of *Aspergillus niger* strains N402 and ATCC 1015. Genome comparison is plotted in circos format (Krzywinski et al., 2009). Arrow indicates a 50.3 kb region present in chromosome 3 in the N402 and the ATCC1015 genome assembly coding for 21 predicted genes which is repeated on scaffold 101 in the proposed assembly of strain ATCC1015.

A comparison of the N402 genome assembly with the genome assembly of the citric acid producer ATCC 1015 confirmed what already has been suggested based on limited sequence data: strain ATCC 1015 and N402 are nearly identical. Nevertheless, as can be seen in **Figure 2** and **Supplementary Table S2**, there were sufficient differences justifying a RNAseq guided *ab initio* gene prediction. For this, the Braker1 (Hoff et al., 2016) tool was applied in combination with the AUGUSTUS eukaryotic gene prediction software (Stanke et al., 2008) and a mixed pool of N402 RNAseq data, obtained from various growth conditions (Odoni et al., 2017), as training set. The results of the *ab initio* predictions are summarized in **Table 1**. In total, from 11,236 gene models 11,285 transcripts were obtained suggesting that some genes may give rise to alternative spliced isoforms. A comparison of the N402 set of *ab initio* predicted genes with those of ATCC 1015 using a similar RNAseq guided approach (Reid et al., 2014) showed significant overlap between 10,915 predicted gene models with a complete congruity for 9,657 gene models. 318 of the 321 additionally predicted genes were also present in the ATCC 1015 genome assembly. Vice versa, from the 779 additional genes predicted in ATCC 1015 using the method of Reid et al. (2014), all but one were present in the N402 genome draft.

### Functional Analysis of Chromosome 2

The *goxB* locus is located on chromosome 2 (Swart et al., 1990). This is also true for the *goxC* gene encoding GOx and the *catR* gene coding for a secreted catalase. Pfam domain predictions of N402 protein encoding genes (**Supplementary File S3**) revealed that N402 genome assembly codes for three putative lactonase (Pfam 10282) encoding genes. The protein products of two of them, which we named *lctA* (ATCC64974_11650) and *lctB* (ATCC64974_18030), have a predicted signal peptide for secretion and are located on chromosome 2. Additionally, they share more than 50% sequence similarity with a recently biochemically characterized and secreted aldonolactonase from *Penicillium oxalicum*, able to catalyze the hydrolysis of D-glucono-1,5-lactone to D-gluconic acid (Peng et al., 2017). Alignment details are provided in **Supplementary Figure S2**. The *lctA* encoding gene (ATCC64974_11650) is found immediate upstream of the *goxC* encoding gene (ATCC64974_11640) and divergently transcribed.

### Identification of the *goxB* Mutation

Paired end Illumina genome sequence reads with a genome coverage of 15 were obtained from the *goxB* strain NW103. By mapping the NW103:*goxB21* sequence reads to the N402 reference genome, potential regions of genomic variation could be observed with multiple chromosomes (see **Supplementary Table S3** for details). However, since the two *goxB* mutants under study are mapped on chromosome II (Swart et al., 1990), protein coding regions of chromosome II were further investigated. One particularly convincing mapped mutation (see **Supplementary Table S3** for details) suggested that the *goxB* locus might be tightly linked to a thioredoxin reductase (TrxR) encoding gene (ATCC64974_16500). TrxR is a flavoprotein with a catalytic selenocysteine residue and part of the two-component thioredoxin system. The thioredoxin system is responsible for maintaining a reducing intracellular environment by controlling the redox potential together with glutathione (GSH) and glutaredoxins. Apart from its function to reduce oxidized Trx, TrxR may also reduce other target proteins (Holmgren and Bjornstedt, 1995; Arnér and Holmgren, 2000).

**FIGURE 3:**
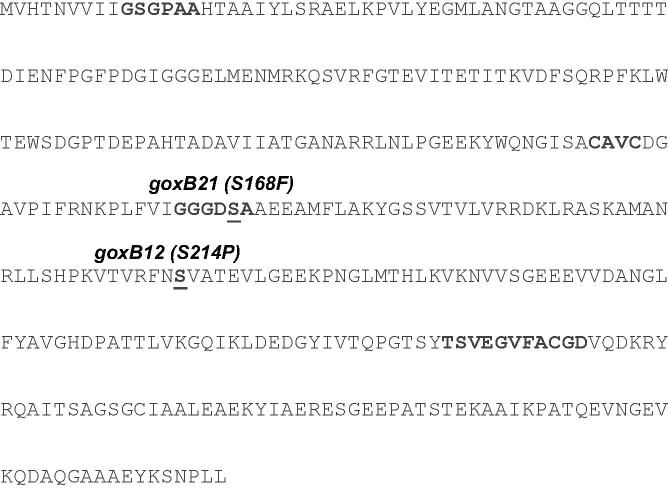
Missense mutations observed in the amino acid sequence of *A. niger* TrxR from two independent *goxB* strains. Missense mutations are indicated in parentheses. Three essential domains can be distinguished in TrxR: a redox-active cysteine pair (CAVC), NADPH-binding domain (GGGDSA), and the FAD-binding domain (GSGPAA and TSVEGVFACGD). Induced mutations are underlined.

From translation start to stop, the *A. niger* N402 *trxR* locus encompasses 1,243 nt and this includes two intron regions. The coding sequence translates into a 367 amino acids protein sequence. To confirm a tight linkage of *goxB locus* to the *trxR* gene, the genome sequences of *trxR* genes from *goxB* strains NW102:*goxB12* and NW103:*goxB21* were subjected to PCR amplification and Sanger sequencing. The results indicated that due to a mutation in NW102:*goxB12*, the serine residue present at position 214 was altered to proline while, consistent with the genome mapping results, in NW103:*goxB21* the amino acid at position 168 was changed from serine to phenylalanine (**Figure 3**).

### Deletion of *trxR* Induces the *goxB* Phenotype

**FIGURE 4:**
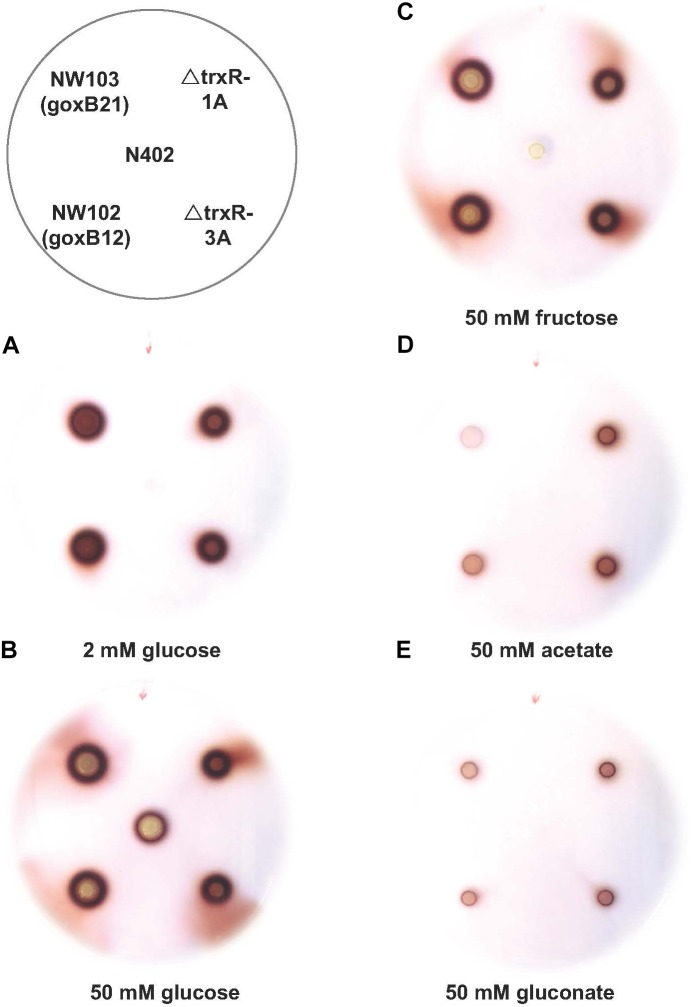
Phenotypic characterisation of trxR gene knock-out strains. Glucose oxidase plate assay of *A. niger* strain N402, NW102:*goxB12*, NW103:*goxB21*, Δ*trxR*-1A, and Δ*trxR*-3A on various carbon sources: **(A)** 2 mM glucose, **(B)** 50 mM glucose, **(C)** 50 mM fructose, **(D)** 50 mM acetate, and **(E)** 50 mM gluconate.

The forward genetics approach pinpointed to either a dysfunctional or nonfunctional thioredoxin reductase as cause of the *goxB* phenotype. To understand the molecular consequences of a *trxR* deletion, the *trxR* gene was replaced by the selection marker *pyrG* from *A. oryzae*. Two independent Δ*trxR* strains labeled Δ*trxR-1A* and Δ*trxR- 3A* were selected for further study. First, a GOx plate assay was used to monitor GOx activity of the wild type, the two *goxB* mutants and the two Δ*trxR* strains under normal GOx inducing and non-inducing conditions. The wild type control, the two mutants and the two Δ*trxR* strains were grown on agar plates containing the following previously tested carbon sources: glucose, fructose, acetate, and gluconate. Compared to the wild type strain the knock-out strains showed a slightly reduced l growth and a normal morphology. Colonies were stained for GOx activity 40 h after incubation (**Figure 4**). Demonstrating the normal GOx activity patterns under these conditions (Swart et al., 1990) the wild type strain N402 colored noticeably only when grown on a 50 mM glucose plate but no visual GOx staining could be observed with 2 mM glucose as carbon source. Likewise, no GOx activity was found when N402 was grown with other carbon sources, while the two Δ*trxR* strains successfully reproduced the *goxB* phenotype: a high level of GOx activity at high and at low glucose concentration as well as under GOx non-inducing conditions, using either fructose, acetate or gluconate as carbon source. From studies with *S. cerevisiae*, it is known that lack of the thioredoxin system blocks sulfate assimilation (Draculic et al., 2000) suggesting that the (slight) reduction in growth observed for the Δ*trxR* strains could be overcome by adding an organic sulfur source. Supplementing solid assay medium with 5 mM methionine indeed had a positive effect on growth of the Δ*trxR* strains but there was no effect on the *goxB* strains indicating that indeed both *goxB* mutants have a dysfunctional rather than a nonfunctional thioredoxin reductase (**Supplementary Figure S5**).

### Transcriptional Response of *goxC, lctA, and catR* in Oxidative Stress Conditions

**FIGURE 5:**
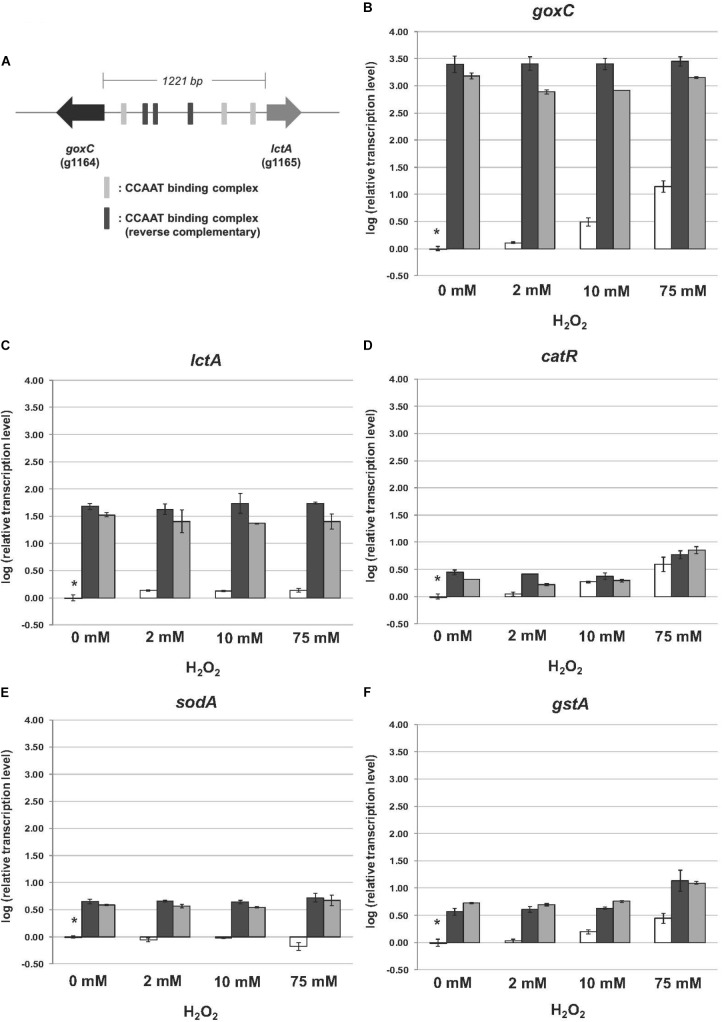
Expression of the glucose oxidase, lactonase, catalase, superoxide dismutase, and glutathione-S-transferase encoding genes upon hydrogen peroxide addition. Samples were taken 3 h after mycelium transfer to minimal medium with different concentrations of hydrogen peroxide (0–75 mM). **(A)** Genomic organization of *goxC* and *lctA* on chromosome II. The six putative CCAAT boxes are indicated in gray. **(B–F)** Expression analysis performed by RT-qPCR with the primers specific for *goxC* **(B)**, *lctA* **(C)**, *catR* **(D)**, *sodA* **(E)**, and *gstA* **(F)** gene. White bars represent expression levels obtained in strain N402, black bars in Δ*trxR*-1A and gray bars in Δ*trxR*-3A. Normalization of the expression data was done using the histone 4-like gene transcript. Results were calculated as relative transcript ratio in logarithmic scale (log10) with means of two biological replicates. Asterisks represents transcript level of the reference sample.

In the GOx mediated bioconversion of glucose to D-glucono-1,5-lactone, hydrogen peroxide is formed in equimolar amounts. Witteveen et al. (1993) observed that in the absence of glucose, addition of hydrogen peroxide to the medium resulted in the induction of GOx, lactonase, and catalase activities in the wild-type strain. Hydrogen peroxide is not only a non-radical reactive oxygen species (ROS), but can also function as second messenger through reversible H_2_O_2_-induced oxidative activation of thiol proteins, and one role of the thioredoxin system is to reverse H_2_O_2_-induced activation (reviewed by Sies et al., 2017). To determine the effects of the absence of this antioxidant system on the expression of *goxC*, *lctA*, and *catR*, the wild type and knock-out strains were monitored by RT-qPCR in the presence of three different oxidative stress-inducing agents, used to generate ROS: H_2_O_2_, diamide and menadione. Hydrogen peroxide increases the intracellular peroxide (O_2_^2−^) level and menadione generates superoxide anion (O^−^), while diamide is a thio-oxidizing agent resulting in fast oxidation of GSH to glutathione disulphide (GSSG), that leads to a GSH/GSSG redox imbalance. To monitor the physiological response of *A. niger* to the oxidative stress applied, two ROS responsive marker genes were included in the RT-qPCR analysis; *gstA* coding for glutathione-S-transferase A and *sodA*, coding for superoxide dismutase A. In *A. nidulans, sodA* gene expression is responding solely to menadione treatment (Pócsi et al., 2005). Cells were pre-grown with fructose as carbon source. Then, either H_2_O_2_ (**Figure 5**), diamide or menadione (**Supplementary Figure S3**), were added to the medium, and cultures were exposed for 3 h to each of the oxidative agents. **Figure 5** displays the effect of a *trxR* knock-out on the expression of the genes in the normal culture conditions. In absence of oxidative stress; in the two Δ*trxR* strains significantly higher expression levels were obtained for all.

Regarding the *A. niger* response to the presence of increasing concentrations of hydrogen peroxide, we observed different transcriptional responses between the five studied genes in the N402 strain. In the wild type strain, the mRNA levels of *goxC*, *catR*, and the ROS responsive marker gene *gstA* increased along with the increased H_2_O_2_ concentration present in the culture media. The *lctA* gene was also induced in the presence of hydrogen peroxide, but transcriptional levels were not increasing along with the increased H_2_O_2_ concentration present in the culture media. In contrast, the ROS responsive marker gene *sodA*, was not upregulated upon hydrogen peroxide addition, and even showed a certain degree of downregulation in the presence of the high concentration (75 mM) of the oxidizing agent in the N402 strain. Regarding the two Δ*trxR* strains, the expression level of *goxC* and *lctA* showed to be much higher than in the N402 strain, in any of the culture conditions, but no further difference in mRNA accumulation levels were observed between oxidative stress and non-inducing conditions. Deletion of the *trxR* gene also influenced the regulation of *catR* and *gstA*, In the absence of exogenous hydrogen peroxide, *catR* and *gstA* showed, compared to the wild-type, higher transcriptional levels in the Δ*trxR* strains. The absence of *trxR* also influenced the regulation of *catR* and *gstA*, which also showed higher transcriptional levels in the mutant strains. However, in the Δ*trxR* strains their expression was still further increased in the presence of 75 mM of hydrogen peroxide.

The presence of diamide (1.8 mM) and menadione (0.8 mM) in the media also had an effect in the transcriptional response of the studied genes in the N402 strain. All of them were transcriptionally activated in the presence of both oxidative agents (**Supplementary Figure S3**). In response to diamide and menadione the *goxC* gene showed a similar response to that observed in the presence of 75 mM hydrogen peroxide, while *lctA* showed higher expression levels with diamide and menadione. The *gstA* gene had a similar transcriptional response in the presence of 75 mM H_2_O_2_ and diamide, but as previously reported (Pócsi et al., 2005), higher transcriptional levels were achieved in the menadione condition. The mRNA accumulation levels of *catR* showed some variation between the three oxidative conditions, and showed a higher transcriptional response in the menadione condition too. The *sodA* gene showed an upregulation in expression in response to menadione, and in contrast to the response in *A. nidulans*, also responded to diamide. Regarding the response of the Δ*trxR* strains to the oxidative stress produced by diamide and menadione, *goxC*, and *lctA* continued to be clearly upregulated when compared to their expression in the reference strain. This was also the case for *catR* and *gstA* in the presence of diamide. However, the expression of these two latter genes in the menadione condition was at the same level in the N402 and Δ*trxR* transformants.

### Transcriptional Analysis of *goxC*, *lctA*, and *catR* in the Presence of Different Carbon Sources

Mycelium transfer experiments were used to study carbon source induced transcriptional changes of *goxC*, *lctA*, and *catR*. Mycelium was pre-grown in sorbitol and transferred to cultures containing minimal medium supplemented with either a low (2 mM) or high (50 mM) concentration of glucose, fructose (50 mM), acetate (50 mM), gluconate (50 mM), and sorbitol (100 mM). Samples for RT-qPCR analysis were taken 4 h after mycelium transfer.

**FIGURE 6:**
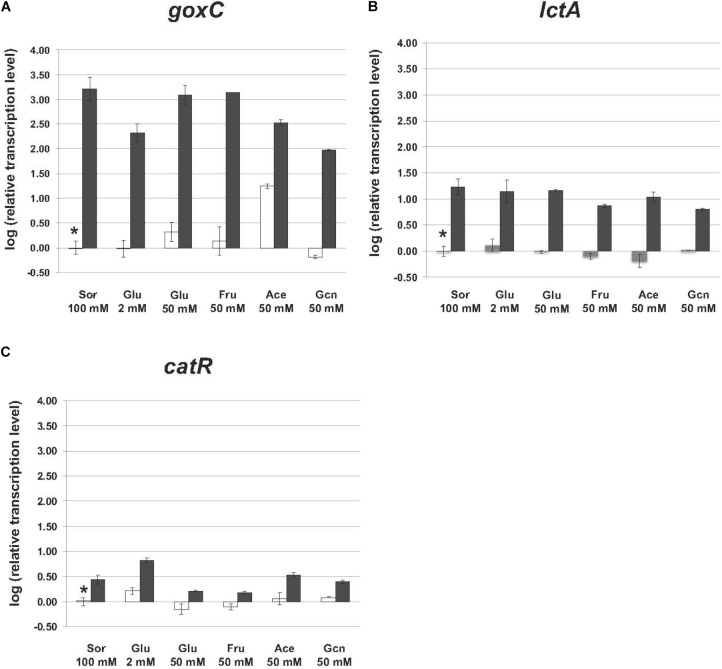
Expression of the glucose oxidase and lactonase, encoding genes of *A. niger* strain N402 and Δ*trxR*-1A in various carbon sources. Samples were taken 4 h after mycelium transfer to the medium with different carbon sources including glucose (Glu), fructose (Fru), acetate (Ace) gluconate (Gcn), and sorbitol (Sor). Expression analysis was performed by RT-qPCR with the primers specific for *goxC* **(A)**, *lctA* **(B)**, and *catR* **(C)** gene. White bars indicate expression levels obtained in strain N402, black bars in Δ*trxR*-1A and gray bars in Δ*trxR*-3A. Normalization of the expression data was done using the histone 4-like gene transcript. Results were calculated as relative transcript ratio in logarithmic scale (log10) with means of two biological replicates. Asterisks represents transcript level of the reference sample.

In the N402 strain, the transcript levels of the *goxC* gene were significantly increased upon a carbon source shift to 50 mM acetate, and in 4 h only slightly induced when the carbon source was shifted to 50 mM glucose. Upon a shift to 50 mM fructose and 2 mM glucose, *goxC* transcript levels remained similar to those observed in the reference condition (**Figure 6**). In addition, *goxC* transcript levels showed to be slightly downregulated in the gluconate condition. No significant changes in expression level were observed for *catR* and *lctA*.

In agreement with the result observed in the GOx plate activity assays, in the Δ*trxR* strain, *goxC, and lctA* transcriptional levels remained highly upregulated in all carbon conditions studied. This was also the case for *catR*, which showed higher mRNA levels in the mutant strains.

## Discussion

The power of forward genetics is that it starts with a phenotype of interest. In an unbiased genetic screen Swart et al. (1990) identified nine independent loci on at least four different chromosomes, all interfering with the normal pattern of GOx expression. While GOx functions outside the cell, and is therefore uncoupled from the internal metabolism, seven GOx complementation groups have in common that they lead to unregulated GOx activities suggesting that GOx expression is complex, involving multiple factors and apparently tightly regulated. As the molecular basis of none of these factors was known, a mapping by sequencing approach was employed. In principle, next-generation sequencing provides a powerful way to efficiently identify artificially induced mutations in genomes. However, a statistically relevant identification of possible nucleotide mutations requires a high-quality genome draft of the immediate parent strain, and given the inherent base-calling error rates of high-throughput sequence mapping data (Quail et al., 2012), defined loci to efficiently search for such mutations. While the genetic criteria have been met, a high-quality genome draft of the immediate parent was not available and was therefore obtained using current standard procedures; a hybrid assembly strategy, mixing long, and short reads of sufficiently high coverage.

### Comparison of ATCC 64974 (N402) and ATTC 1015 Whole Genome Assemblies

The newly obtained genome assembly from the N402 strain was compared with the nearest published genome sequence of *A. niger* strain ATCC 1015 (Andersen et al., 2011). Strain N402 is a descendent of the wild type strain N400 and frequently used as a laboratory strain as it carries the *cspA1* mutation making it more suitable for laboratory manipulations. Overall, there is very high level of sequence identity (**Supplementary Table S2**). Having said that, we also observed a 50 kb duplication in the ATCC 1015 assembly between chromosome 1 and 3 not present in the N402 assembly (**Figure 2**), single and multiple, consecutive nucleotide differences, and multiple small gaps and insertions (**Supplementary Table S2**). Note that for the two strains different strategies were used for genome sequencing and assembly and therefore many of these differences may be due to technical issues.

Although, not the aim of this study, with such a high level of sequence identity, it is tempting to speculate on the possible genomic locations of *loci* that may contribute to the *cspA1* phenotype. In that respect, the most promising difference between the ATCC 1015 wild type strain and the N402 *cspA1* strain is an ∼9 kb deletion in N402 scaffold 301, at nucleotide position 1.037.003. The deletion in the N402 assembly is responsible for deleting the 3-prime half of gene ATCC64974_3000 (incomplete N402 version) encoding the C-terminal half of a 1641 amino acid protein (Pubmed protein_id EHA24670) harboring three kinesin associated Pfam domains (pfam00225; pfam12473; pfam16183). Kinesin family proteins have important roles in intracellular transport and in cell division. In aspergilli, the encoded gene is ubiquitous present and appears to be a single copy gene. Additionally, the 9 kb deletion in strain N402, deletes a gene coding for a hypothetical protein (Aspni3p7_00651; corresponding protein id CAK42436.1) that seems to be unique for *A. niger*. It should be noted that two rounds of low dosed UV mutagenesis and selection were required to obtain the *cspA1* phenotype. While, based on our N400 Illumina genome sequence data, the 9 kb sequence gap is non-existent in the N400 parental strain, and the kinesin like protein appears to be a promising candidate, other less obvious mutations may play an equally important role.

### A Model for GOx Regulation

Regarding *goxB*, previous studies indicated that *A. niger* strains carrying mutations in this locus (NW102:*goxB12* and NW103:*goxB21*) resulted in constitutive expression, modulated to some extent by the carbon source, of three enzyme activities: GOx, lactonase and catalase (Witteveen et al., 1990, 1993). In a forward genetic screen the *goxB21* locus in NW103 could be linked to a thioredoxin reductase coding gene (*trxR*). Missense mutations in this gene, which could compromise the TrxR enzyme function, were found in both *goxB* strains.

The thioredoxin system of *A. nidulans*, composed thioredoxin (TrxA) and thioredoxin reductase (TrxR) has been biochemically fully characterized (Thön et al., 2007). The role of TrxA is to reduce other proteins by cysteine thiol-disulfide exchange. The oxidized form of TrxA is subsequently reduced by TrxR using NADPH as electron donor and its own redox-active cysteine pair, and FAD as cofactor. *A. niger* TrxR shows 85% sequence identity with *A. nidulans* TrxR and 65% identity with the two yeast thioredoxin reductases. In NW103:*goxB21*, the mapped mutation changed the polar serine residue 168 into an aromatic and much larger phenylalanine residue.

A multiple alignment of *A. niger* N402 TrxR with a selection of other low molecular weight fungal thioredoxin reductases (**Supplementary Figure S4**) indicated that Ser168 is strongly conserved and part of the NADPH-binding domain (GGGDSA) which suggests a direct interference with NADPH binding. Although, the *goxB12* and *goxB21* mutations have a similar phenotype for GOx expression the impact of the Ser214Pro mutation is less clear. There is a lower level of sequence conservation for this particular region and the particular serine can also be threonine. We further investigated the consequences of this mutation at structural level by evaluating the equivalent position in the available yeast structure (Oliveira et al., 2010) (**Supplementary Figure S4**) which suggested that changing serine 214 into a more rigid proline may have serious structural consequences.

TrxA and TrxR comprise a thioredoxin system. While the *goxB/trxR* mutations in *A. niger* NW102 and NW103 did not show an severe growth phenotype (**Supplementary Figure S5**), a *trxA* deletion in *A. nidulans* showed a decreased growth, an inability to form reproductive structures and a high sensitivity toward added hydrogen peroxide (Thön et al., 2007). To exclude that the *goxB* mutations represented a dysfunctional rather than a nonfunctional TrxR, two independent *trxR* knock-out strains were obtained and used (Δ*trxR-1A* and Δ*trxR-3A*) to study the knock-out phenotype. Once the deletion of *trxR* gene was confirmed in the Δ*trxR-1A* and Δ*trxR- 3A* strains, both of which showed no strong growth phenotype, a GOx activity plate assay confirmed that the Δ*trxR* strains could successfully reproduce the *goxB* phenotype. By comparing on agar plates, growth of the *goxB* mutant strains with the Δ*trxR* strains, both mutants appear to express a dysfunctional rather than a nonfunctional TrxR. Upon supplementation with 5 mM methionine, growth of the knock-out strain was stimulated and almost similar to the wild type strain N402 while we did not observe such a growth defect for the mutants. Upon deletion of TrxA in *A. nidulans* (Thön et al., 2007) could restore the growth defect by adding glutathione. We also tested glutathione and somewhat surprising there was no restoration of growth, however in all strains a delay of spore formation was observed (**Supplementary Figure S5**). Although, TrxR and TrxA are the two parts of the thioredoxin system, a direct comparison between a Δ*trxA* in *A. nidulans* (Thön et al., 2007) and Δ*trxR* in *A. niger* is difficult.

In order to further understand the pivotal role of TrxR in GOx regulation, a series of transcriptional analyses of *goxC*, *lctA*, and *catR*, relevant for the biotransformation of glucose to gluconic acid and the fungus oxidative stress response, were performed. In the absence of oxidative stress, these genes showed a basal expression level in the N402 strain. However, all of them, were upregulated in the Δ*trxR* mutants, indicating a prominent role of the thioredoxin/thioredoxin reductase redox system in their transcriptional regulation. To study the role of the carbon source in regulation of expression of the GOx system carbon source shift experiments were used. In contrast to the immediate effect elicited by hydrogen peroxide addition, on the short term the expression level of the *goxC* gene was not significantly induced upon a substrate shift from sorbitol to glucose and most other carbon sources in the N402 strain. A shift to 50 mM acetate as carbon source, however, resulted in a 10-fold upregulation of the *goxC* gene expression. In yeast, acetate has been described as an agent able to produce oxidative stress involving the Yap1p transcriptional regulator (Semchyshyn et al., 2011).

The absence of the *trxR* gene affected strongly the expression of the *goxC* gene, in all carbon conditions tested. The gene was highly upregulated in the presence of sorbitol, both glucose concentrations, fructose, and gluconate, with differences in mRNA accumulation between N402 and Δ*trxR* strains of around 2–3 orders of magnitude. The results obtained confirmed that the *goxB* mutation and knock-out (Δ*trxR*) results in a constitutive expression of GOx, lactonase and catalase, although this may be modulated to some extent depending on the carbon source.

**FIGURE 7:**
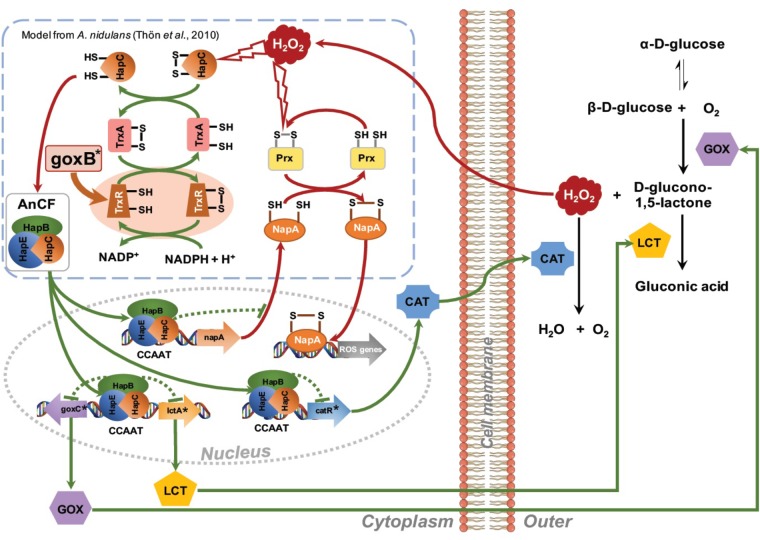
Proposed network for regulation of the glucose oxidase system. Amplification of the H_2_O_2_ signal is achieved via a positive feedback loop. H_2_O_2_ induced oxidative modification of thiol groups in ROS sensitive regulatory proteins such as AnCF complex (Thön et al., 2010) or NapA (Marinho et al., 2014) leads to activation of *goxC* expression. Outside the cell an increase GOx activity may lead to a further increase in turnover of glucose in gluconic acid and H_2_O_2_. The increased extracellular H_2_O_2_ concentration is counterbalanced by H_2_O_2_ induced expression of the *catR* gene, leading to an extracellular catalase activity. Simultaneously, in a negative feedback loop, ROS activation of the thioredoxin system facilitates a return to the native state. GOx specific genes are indicated with an asterisk.

The presence of six CCAAT sequences present in the 1,221 bp intergenic region between the *goxC* and *lctA* gene (**Figure 5A**) and the direct involvement of H_2_O_2_, the second product of the conversion of glucose to D-glucolactone as signaling molecule for induction of the GOx system, supports a model of regulation of the GOx system involving positive and a negative feedback loops. Signal amplification is achieved via a positive feedback loop in which H_2_O_2_ targets the CCAAT-binding factor (AnCF) (Thön et al., 2010) and/or YAP-like basic leucine zipper (bZIP) transcription factor (NapA) (Marinho et al., 2014). In either case, H_2_O_2_ induced oxidative modification of thiol groups in these proteins leads to activation of *goxC* expression possibly through derepression (AnCF) and/or activation (NapA) (Thön et al., 2010). Simultaneously, in a negative feedback loop, ROS activation of the thioredoxin system leads to reactivation of AnCF activity and inactivation of NapA activity (Thön et al., 2010). In the presence of a sufficient supply of oxygen and glucose, increased GOx activity will lead to an increased turnover of glucose in gluconic acid and H_2_O_2_. Outside the cell, the effect of this positive feedback loop is counterbalanced by H_2_O_2_ induced expression of the *catR* gene. The *catR* encodes an extracellular catalase and a single CCAAT box is found 633 bp upstream of the *catR* start-site. Independent from this mechanism, the thioredoxin system is involved in the redox regulation of the AnCF/NapA controlled transcriptional activity, thereby making it a monostable system (**Figure 7**).

The proposed model can explain the *goxB* phenotype and, the requirements of a sufficient amounts of oxygen and a high glucose concentration for induction of the GOx system. It should be noted, however, that this model is highly speculative and lacks many details on the exact nature of the element(s) involved in the regulation of *goxC in A. niger*. Along with *goxC*, ROS defense systems appear to be upregulated in the two *goxB* knockout strains (**Figure 5**). Currently it is, unclear whether this due to insufficient amount of reduced TrxA or results from a direct involvement of the compromised thioredoxin system in AnCF /NapA controlled transcriptional activity.

## Author Contributions

TL and PS conceived and designed the work. TL performed the experiments. TL, BN, and PJ analyzed the data. TL, BN, JT-R, and PS contributed to the interpretation of the data. TL and PJ wrote the manuscript. TL, JT-R, and PS critically revised the manuscript for intellectual content. All authors have read and agreed to the submission of the manuscript.

## Conflict of Interest Statement

The authors declare that the research was conducted in the absence of any commercial or financial relationships that could be construed as a potential conflict of interest.
