# Supplementary material for: Forward Genetics by Genome Sequencing Uncovers the Central Role of the Aspergillus niger goxB Locus in Hydrogen Peroxide Induced Glucose Oxidase Expression
Source: Front Microbiol. 2018 Sep 24;9:2269. doi: 10.3389/fmicb.2018.02269 (PMC6165874; doi:10.3389/fmicb.2018.02269)
Supplement: Supplementary file 1 [file Data_Sheet_1.PDF]

Supplementary Material

Forward genetics by genome sequencing uncovers the central role of the *Aspergillus niger goxB* locus in hydrogen peroxide induced glucose oxidase expression

Thanaporn Laothanachareon[1,2], Juan Antonio Tamayo-Ramos[3], Bart Nijsse[1], Peter J. Schaap[1*]

*** Correspondence:** Peter J. Schaap: peter.schaap@wur.nl (PJS)

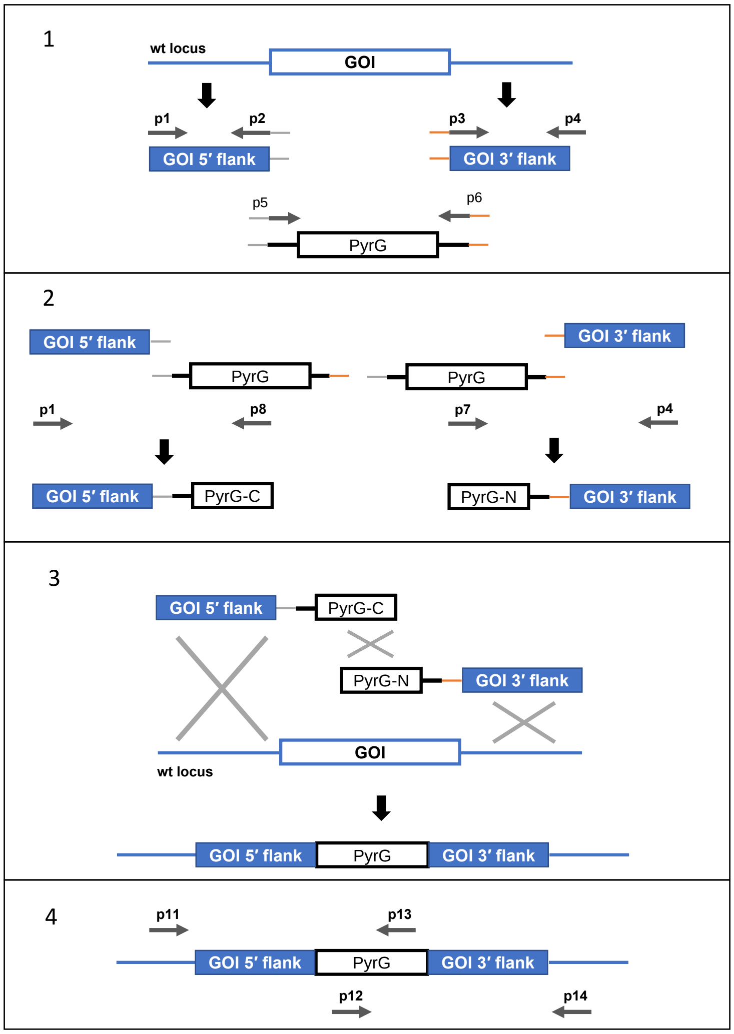

Supplementary Figure 1. Scheme representing experimental steps for deletion of *trxR* from the *A. niger* genome. 1) The 5' flanking region of the *trxR* gene was amplified with primers trxR_KO_5'flank_FW_p1 and trxR_KO_5'flank_RV_p2. The 3' flanking region of the *trxR* gene was amplified with primers trxR_KO_3'flank_FW_p3 and trxR_KO_3'flank_RV_p4. The *pyr*G gene was amplified with primers AOpyrG12FW_p5 and AOpyrG13RV_p6. 2) Deletion of the *trxR* gene by the

split marker method consisting of two overlapping DNA fragments to disrupt of the *trxR* gene. Fragment one contains the 5' flank of the *trxR* gene and a partial version of the *pyrG* gene and was constructed by primers trxR_KO_5'flank_FW_p1 and AOpyrG15RV_p8. Fragment two contains an overlapping partial version of the selection marker and the 3' flank of the *trxR* gene and was established by using primers AOpyrG14FW_p7 and trxR_KO_3'flank_RV_p4. 3) Deletion of the *trxR* gene by the split marker method using the two overlapping DNA fragments and transformation in *A. niger* strain MA169.4. 4) Confirmation of the integration position. The trxR_KO_5'intcheck_ FW_p11 and AOpyrG_KO_intcheck_RV_p13 and the AOpyrG_KO_intcheck_FW_p14 and trxR_KO_3'intcheck_RV_p12 were applied to check on the 5′ flanking and the 3′ flanking region, respectively. The gene replacement and the purity of the knock-out strains were also checked (scheme does not show). The AOpyrG13RV_p6 and AOpyrG13RV_p6 primers were used to determine the *pyrG* marker gene replacement and the trxR_FW and trxR_RV primers were verified the purity of the knock-out strains.

A

```
P.oxalicum_AltA      MRLTHAPLFSLLTLTGASLQDSAGRHTTSRLWATHYNGNVYSLAFNG-----KSLSLTDT 55
A.niger_N402_LctA    MK------FSVV-----PLLFGATSAVASRLYAASYAGTVTTLSLSQSSKGHYELETVAQ 49
A.niger_N402_LctB    ----MGRHFALS-----WLATCLPLATATNLYATHYDGSVYSLSLQRS-DDTYSLSIASS 50
                             *::       *       .::.*:*: * *.* :*::.       .*. .

P.oxalicum_AltA      AKTCGTMPSWLTFDRDTQTVYCSDEAGSADPSTHGSLWAYHAGRDGSLQQYAKTDTVGGG 115
A.niger_N402_LctA    STDCGTNPSWLMLDHDNRVLYCLDEAVD---LANGTLTSFAIRPNGTLSKVVQLETIAGP 106
A.niger_N402_LctB    LKTCGSMPSWLTFDSASRILYCSDESGD--ASTNGSLTTLAADEDGTLTEIATAAAPGGG 108
                      . **: **** :*  .: :** **: .    ::*:* :     :*:* : .   : .*

P.oxalicum_AltA      VNSVIYKND--QGDKYLAIAHYEGSAVSTFALPLHDG-DSALQEFHFKLTRPGAVA-QQD 171
A.niger_N402_LctA    VMSQFYSAPGVPHRKFFAVAHYEGSAVTSYSLDPISGVFNRSQTFTYTLPAPGPVADRQD 166
A.niger_N402_LctB    VNSIFYSGD--DGTQYLAIAHYGGSAMSTFRLPLNQG-DENLQVFRYTLSETKQNP-QQN 164
                     * * :*.       :::*:*** ***:::: *   .*  .  * * :.*        :*:

P.oxalicum_AltA      SPHPHEVFLDPTGSFVVSPDLGADLLRIYSIDGESGKLH-ECPSVNITFGFGPRHGVFWS 230
A.niger_N402_LctA    APHSHGVVVDPTGQFVLVPDLGADLIRIFHINPSTGLLEPQAPLVA-APGSGPRHGVFWT 225
A.niger_N402_LctB    APHPHQVLLDPTGSFILVPDLGADLVRVYAIDKSSGELDGTCPDLSYPEGSGPRHGLFWQ 224
                     :** * *.:****.*:: *******:*:: *: .:* *.  .* :    * *****:**

P.oxalicum_AltA      DGTSDQKHSRDDQTGHASDSRQAQAVGKTMMYLANEIGGTVDVFDVTYARSGCLSFEKIQ 290
A.niger_N402_LctA    PAGTTAKQA--------------KHEDVIFYLTSELNNHVTGYRVTYPSNGTISFTEFY 270
A.niger_N402_LctB    TEHSTLTRQL-----------KTRQQDSQVLYIVDELDGHFKGFTVSYTSDGCLGFDEFQ 273
                        :  .:                   . ::*:..*:.. .  : *:*  .* :.* ::

P.oxalicum_AltA      TMVPYTNGVMPKGATPAEIRL--IDDTLYVSIRSDQGFAPSDSMVALD-----RSPRTGT 343
A.niger_N402_LctA    TANSYGGAVPPNGSKVAEIAISPQNNRLVVSNRDDNTFGTNNDSIAVFNCADESGSHPTN 330
A.niger_N402_LctB    SFEPYTDGQLPDGATPSEIRQ--AGNSLYVSIRSDQGFAPNDSMANLD-----HSSN-NT 325
                     :   * ..  *.*:. :**     .: * ** *.*: *. .:.   :       . .  .

P.oxalicum_AltA      VKFRQKTPSYGKVPRTVAINRAGDLVAIGDQASSNVAIVRRDRKTGNLGEKIATLQVGEP 403
A.niger_N402_LctA    VSFGGLYPAYGSSPRQFEMSARNEMIAEALQNTHAVGVTQWNGKTGKPVPLVAKK----- 385
A.niger_N402_LctB    ITLNELTSSYGTVPRTFVINQAGDLVAIGDQSSSNVAIVARDPQTGKLGDEVANVQIGEP 385
                     :.:     :**. **  . :.  .:::* . * :  *.:.  : :**:    :*.

P.oxalicum_AltA      GQVGTAEGLSSVIWDE 419
A.niger_N402_LctA    ---SLVGEVVSVVWDE 399
A.niger_N402_LctB    GVVGTSTGLSSVVWDE 402
                        .    : **:***
```

B

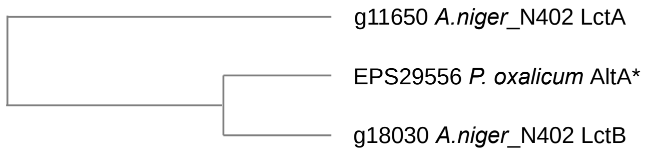

Supplementary Figure 2. Alignment of *Aspergillus niger* lactonase LctA and LctB and *Penicillium oxalicum* aldonolactonase AltA. Amino acid sequences were aligned by Clustal Omega (A) and the phylogenetic tree of three protein were constructed (B).

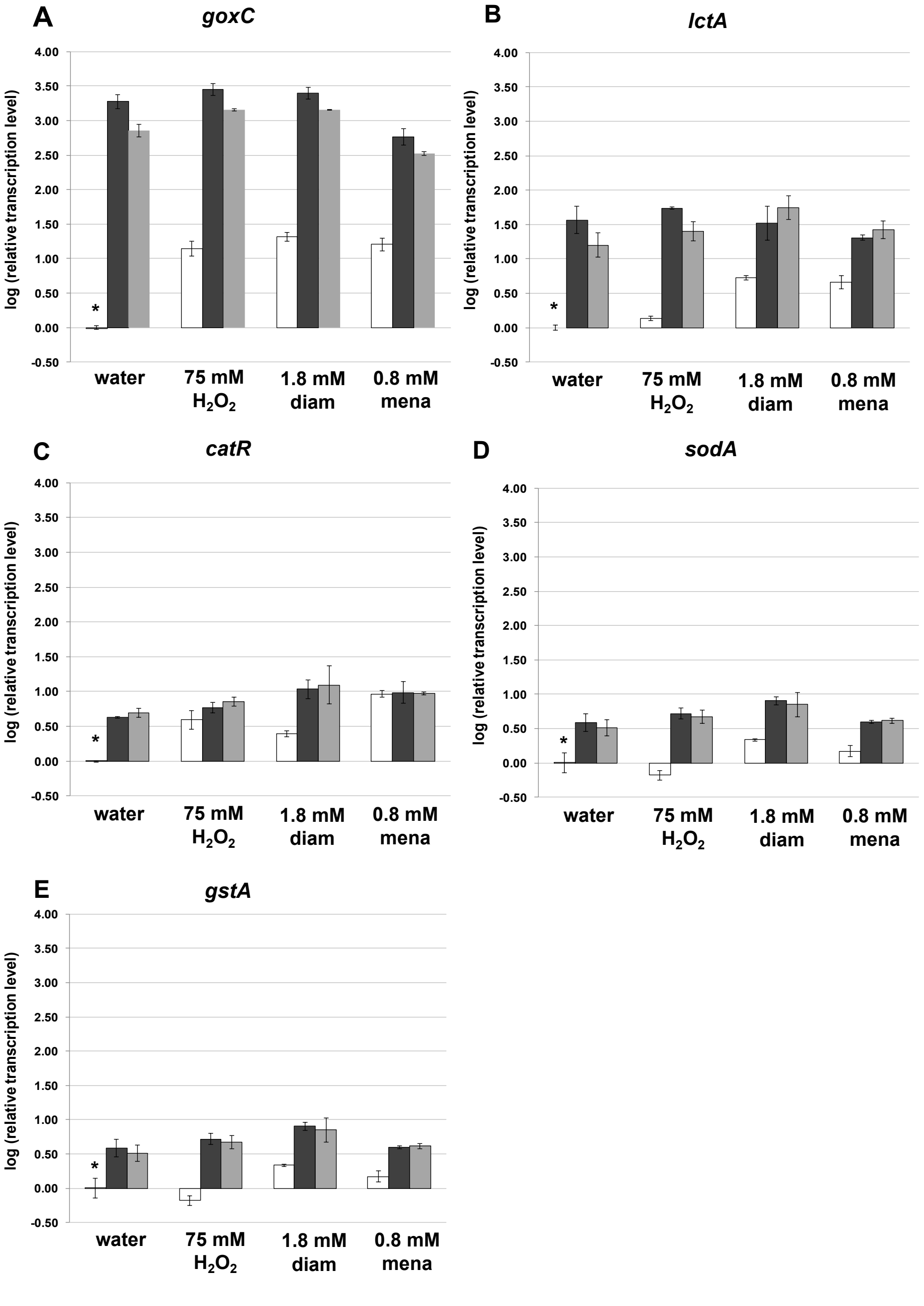

Supplementary Figure 3. Expression of the glucose oxidase, lactonase, catalase, superoxide dismutase and glutathione-S-transferase encoding genes upon oxidizing agents. Samples were taken 3 hours after mycelium transfer to minimal medium with different oxidizing agents: 75 mM hydrogen peroxide (H_2O_2), 1.8 mM diamine (diam) and 0.8 mM menadione (mena). Expression analysis was performed by RT-qPCR with the primers of *goxC* (A), *lctA* (B), *catR* (C), *sodA* (D), and *gstA* (E) gene. Normalization of the expression data was done using the histone-like gene "*hist*" transcript (gene ID 207921). Results were calculated as relative transcript ratio in logarithmic scale (log) with means of two biological replicates. An asterisk represents transcript level of the reference sample. The white bar represented *A. niger* N402, the black was Δ*trxR*-1A and the grey was Δ*trxR*-3A. The data was shown in Supplementary file 4.

A

```
S.cerevisiae          MVHNKVTIIGSGPAAHTAAIYLARAEIKPILYEGMMANGIAAGGQLTTTTEIENFPGFPD  60
T.reesei              -MHTKVVVIGSGPAAHTAAIYLARAELKPVLFEGFMANGTAAGGQLTTTTEIENFPGFPK  59
P.chrysogenum         MVHSKVVIIGSGPGAHTAAIYLSRAELQPVLYEGMLANGTAAGGQLTTTTDVENFPGFPS  60
A.niger_N402          MVHTNVVIIGSGPAAHTAAIYLSRAELKPVLYEGMLANGTAAGGQLTTTTDIENFPGFPD  60
A.niger_CBS513.88     MVHTNVVIIGSGPAAHTAAIYLSRAELKPVLYEGMLANGTAAGGQLTTTTDIENFPGFPD  60
A.nidulans            MVHSKVVIIGSGPAAHTAAIYLSRAELKPVLYEGMLANGTAAGGQLTTTTDVENFPGFPD  60
A.terreus             MVHNKVVIIGSGPAAHTAAIYLSRAELQPVLYEGMLANGTAAGGQLTTTTDIENFPGFPD  60
                       :*.:*.:*****.********:***::*:*:**::*** **********::*******.

S.cerevisiae          GLTGSELMDRMREQSTKFGTEIITETVSKVDLSSKPFKLWTEFNEDAE---PVTTDAIIL 117
T.reesei              GIMGQELMDNMRAQSERFGTEIITDTVTTLDLSSRPFKFSTEFNPD----ETHTADAVII 115
P.chrysogenum         GIGGAELMDNMRAQSERFGTEIITETISKLDLSSRPFKMWTEWNDDEGSEPVRTADAVII 120
A.niger_N402          GIGGGELMENMRKQSVRFGTEVITETITKVDFSQRPFKLWTEWSDGPTDEPAHTADAVII 120
A.niger_CBS513.88     GIGGGELMENMRKQSVRFGTEVITETITKVDFSQRPFKLWTEWSDGPTDEPAHTADAVII 120
A.nidulans            GIGGSELMDAMRKQSIRFGTEVITETISRVDLSQRPFKLWTEWNDGPDNEPARTADAVII 120
A.terreus             GIGGGELMDNMRKQSIRFGTEVITETISRVDLSQRPFKLWTEWNDGPDNEPARTADAVII 120
                      *: * ***: ** ** :****:**:*:: :*:*.:***: **:. .       *:**:*:

S.cerevisiae          ATGASAKRMHLPGEETYWQKGISACAVCDGAVPIFRNKPLAVIGGGDSACEEAQFLTKYG 177
T.reesei              ATGASARRLNLPGEDKYWQNGISACAVCDGAVPIFRNKPLFVIGGGDSAAEEATFLTKYG 175
P.chrysogenum         ATGANARRLNLPGEETYWQNGISACAVCDGAVPIFRNKPLYVIGGGDSAAEEAMFLAKYG 180
A.niger_N402          ATGANARRLNLPGEEKYWQNGISACAVCDGAVPIFRNKPLFVIGGGDSAAEEAMFLAKYG 180
A.niger_CBS513.88     ATGANARRLNLPGEEKYWQNGISACAVCDGAVPIFRNKPLFVIGGGDSAAEEAMFLAKYG 180
A.nidulans            ATGANARRLNLPGEDVYWQNGISACAVCDGAVPIFRNKPLFVIGGGDSAAEEAIFLTKYG 180
A.terreus             ATGANARRLNLPGEEKYWQNGISACAVCDGAVPIFRNKPLYVIGGGDSAAEEAMFLAKYG 180
                      ****.*:*::****: ***:******************* ********.*** **:***

S.cerevisiae          SKVFMLVRKDHLRASTIMQKRAEKNEKIEILYNTVALEAKGDGK---LLNALRIKNTKKN 234
T.reesei              SHVTVLVRRDQLRASKAMASRLLKHPKVTVRFNTVATEVKGDK--DGLMSHLVVKNVVTG 233
P.chrysogenum         SSVTVLVRKDKLRASNIMADRLLAHPKCKVRFNTVATEVIGENKPNGLMTHLRVKDVLSN 240
A.niger_N402          SSVTVLVRRDKLRASKAMANRLLSHPKVTVRFNSVATEVLGEEKPNGLMTHLKVKNVVSG 240
A.niger_CBS513.88     SSVTVLVRRDKLRASKAMANRLLSHPKVTVRFNSVATEVLGEEKPNGLMTHLKVKNVVSG 240
A.nidulans            SSVTVLVRRDKLRASKAMASRLLANPKVTVRFNTVATEVLGEKKLNGLMTHLRVKNVLTG 240
A.terreus             SSVTVLVRRDKLRASKAMASRLLSHPKVNVRFNTVAVEVLGEQKPMGLMTHLRVKNTVTG 240
                      * * :***:*:****. * .*    : *  : :*:** *. *:     *:. * :*:. ..

S.cerevisiae          EETDLPVSGLFYAIGHTPATKIVAGQVDTDEAGYIKTVPGSSLTSVPGFFAAGDVQDSKY 294
T.reesei              AEETLEANGLFYAVGHDPATGLVKGQVDVDAEGYIITKPGTTETSVPGVFAAGDVQDKRY 293
P.chrysogenum         AEEVVEANGLFYAVGHDPASGLVKGQVELDDEGYIITKPGTSFTNVEGVFACGDVQDKRY 300
A.niger_N402          EEEVVDANGLFYAVGHDPATTLVKGQIKLDEDGYIVTQPGTSYTSVEGVFACGDVQDKRY 300
A.niger_CBS513.88     EEEVVDANGLFYAVGHDPATTLVKGQIKLDEDGYIVTQPGTSYTSVEGVFACGDVQDKRY 300
A.nidulans            EEETLEANGLFYAVGHDPATALVKGQVELDEDGYIATKPGTSFTSVEGVFACGDVQDKRY 300
A.terreus             DEETVDANGLFYAVGHDPATGLVKGQVDLDEDGYILTKPGTSYTSLEGVFACGDVQDKRY 300
                       *  : ..*****:** **: :* **:. *  *** * **:: *.: *.**.*****.:*

S.cerevisiae          RQAITSAGSGCMAALDAEKYLTSLE----------------------------------- 319
T.reesei              RQAITSAGTGCMAALEAEKYITEME----------------------------------- 318
P.chrysogenum         RQAITSAGSGCVAALEAEKFIAETETHQEAKPVL-------------------------- 334
A.niger_N402          RQAITSAGSGCIAALEAEKYIAERESGEEPATSTEKAAIKPATQEVNGEVKQDAQGAAAE 360
A.niger_CBS513.88     RQAITSAGSGCIAALEAEKYIAERESGEEPATSTEKAAIKPATQEVNGEVKQDAQGAAAE 360
A.nidulans            RQAITSAGSGCIAALEAERFIGESESNEEIPPAHANPA----------------L----- 339
A.terreus             RQAITSAGSGCMAALDAEKFIAEHESPEEPAPVIETEK----------------SNTGTE 344
                      ********:**:***:**::: . *

S.cerevisiae          -------      319
T.reesei              -------      318
P.chrysogenum         -------      334
A.niger_N402          YKSNPLL      367
A.niger_CBS513.88     YKSNPLL      367
A.nidulans            -------      339
A.terreus             YKSNPLL      351
```

B

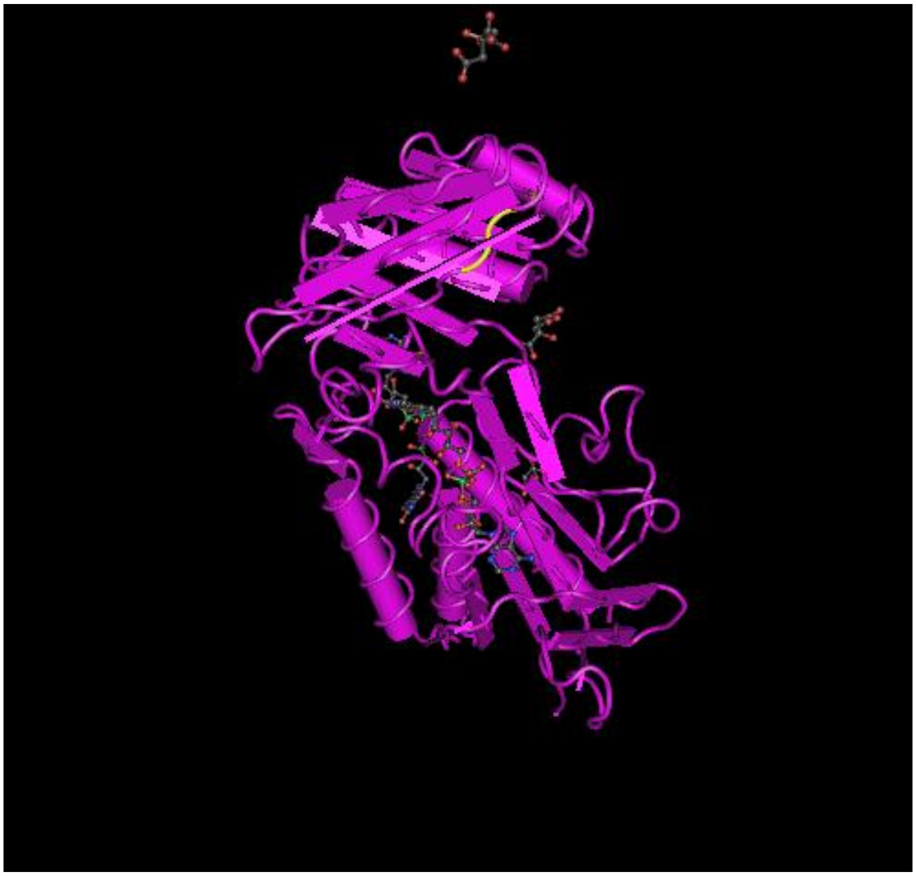

Supplementary Figure 4. A) Multiple alignment of *A. niger* N402 TrxR with a selection of other low molecular weight fungal thioredoxin reductases present in EMBL and SWISSPROT databases. The accession numbers are for *Saccharomyces cerevisiae* (P29509), *Penicillium chrysogenum* (P43496), *Trichoderma reesei* (A0A024S5M9), *A. nidulans* (Q08GE2), *A. terreus* (Q0CT03), and *A. niger* CBS513.88 (A2Q9P0). The FAD binding domain is indicated in blue, the NADPH binding domain in red and the redox-active cysteine pair in green. Amino acid changes in *goxB21* and *goxB12* are indicated with orange and purple triangles respectively . **B)** Crystal Structure of Saccharomyces cerevisiae thioredoxin reductase structure 3ITJ chain_A (Oliveira et al., 2010) highlighting in yellow the equivalent position of the goxB12 S214P mutation in *A. niger* NW102.

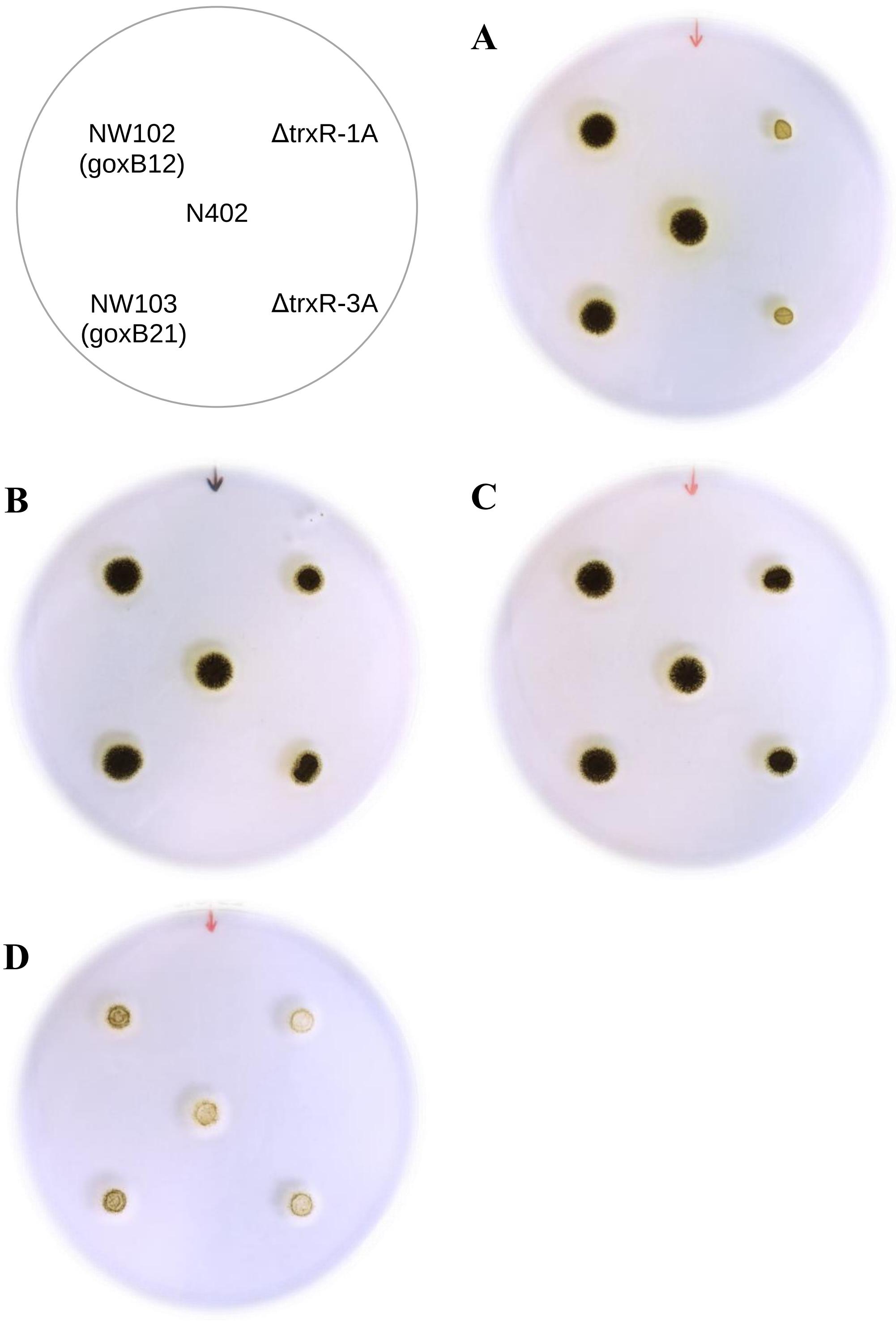

Supplementary Figure 5. Growth of *A. niger* wildtype N402, the mutant strain goxB12 and goxB21 and the knockout strain ΔtrxR-1A and ΔtrxR-3A on solid assay medium **(A)**, supplemented with 5 mM **(B)** or 25 mM **(C)** methionine or 25 mM glutathione **(D)**.
