# Supplementary material for: Forward Genetics by Genome Sequencing Uncovers the Central Role of the Aspergillus niger goxB Locus in Hydrogen Peroxide Induced Glucose Oxidase Expression
Source: Front Microbiol. 2018 Sep 24;9:2269. doi: 10.3389/fmicb.2018.02269 (PMC6165874; doi:10.3389/fmicb.2018.02269)
Supplement: Supplementary file 2 [file Data_Sheet_2.PDF]

Supplementary Material

Forward genetics by genome sequencing uncovers the central role of the *Aspergillus niger goxB* locus in hydrogen peroxide induced glucose oxidase expression

Thanaporn Laothanachareon[1,2], Juan Antonio Tamayo-Ramos[3], Bart Nijsse[1], Peter J. Schaap[1*]

*** Correspondence:** Peter J. Schaap: peter.schaap@wur.nl (PJS)

Table S1. Primers used in this study.

Primer name	Primer sequence (5´ → 3´)
trxR_KO_5'flank_FW_p1	CGATGACGAAGATGATGTGC
trxR_KO_5'flank_RV_p2	CAATTCCAGCAGCGGCTTAGTTTTGTTGCAGGGACGAC
trxR_KO_3'flank_FW_p3	ACACGGCACAATTATCCATCGGCATTGGCTTATGGACTGGT
trxR_KO_3'flank_RV_p4	GAGGTGAAACGGCCCTACTT
AOpyrG12FW_p5	AAGCCGCTGCTGGAATTG
AOpyrG13RV_p6	CGATGGATAATTGTGCCGTGT
AOpyrG14FW_p7	ATTGACCTACAGCGCACGC
AOpyrG15RV_p8	CCGGTAGCCAAAGATCCCTT
trxR_KO_5'intcheck_FW_p11	GAGGAAGATGAGGACGATGG
trxR_KO_3'intcheck_RV_p12	AAGCGCTTGGCCTCATGT
AOpyrG_KO_intcheck_RV_p13	AATTCCACGGCCAGCAATAA
AOpyrG_KO_intcheck_FW_p14	GGCAATTGTCGTGCAGTTTG
trxR_FW	ATGGTGCACACCAACGTCG
trxR_RV	TTAAAGCAGAGGGTTGGACTTGTAC
qPCR_hist_FW	ACAATGACTGGCCGTGGAAAGG
qPCR_hist_RV	ATACGCTTGACACCACCACGAC
qPCR_goxC_FW	AATGCTGCCCGTGTGTATGGTG
qPCR_goxC_RV	TGGGACGACATTTGCGTAGGAG
qPCR_lct_FW	CGGAAATTGCCATCTCACC
qPCR_lct_RV	GGCGATGGAATCGTTGTTG
qPCR_catR_FW	GCTGAAGTTGAACAGGCTGGTTTC
qPCR_catR_RV	TCGGTGAAGTCAATGCCAGGAAC
qPCR_gstA_FW	CCGACATTACCCTGTACACTGC
qPCR_gstA_RV	TCTCGACTTTGTAGGGCAGACC
qPCR_sodA_FW	TGGCAACGAGGAGTCCAAGAAGAC
qPCR_sodA_RV	GCAATGCCAATGACACCGCAAG

Table S2. N402 Assembly Statistics and ATCC 1015 v7 reference statistics

**N402 Assembly Statistics**	**Scaffolds**	**Contigs**
# contigs	19	31
# contigs (>= 0 bp)	19	31
# contigs (>= 1000 bp)	19	29
# contigs (>= 5000 bp)	19	28
# contigs (>= 10000 bp)	19	28
# contigs (>= 25000 bp)	18	25
# contigs (>= 50000 bp)	16	21
Largest contig	5108653	5108653
Total length (>=0)	35570168	35561663
Total length (>=1000)	35570168	35560225
Total length (>=25000)	35552613	35503469
Total length (>=50000)	35478697	35366303
N50	2899611	2870019
N75	2069062	1451243
L50	4	4
L75	8	9
# N's	8512	7
# N's per 100 kbp	23.93	0.02
GC (%)	49.59	49.59
**ATCC 1015 v7 reference statistics**		
# relocations	9	6
# translocations	2	1
# inversions	1	0
# mismatches	1136	1154
# indels	396	395
Indels length	3059	2976
# mismatches per 100 kbp	3.27	3.32
# indels per 100 kbp	1.14	1.14
# indels (<= 5 bp)	267	269
# indels (> 5 bp)	129	126

Table S3. The mutations of NW103:goxB mapped to the N402 sequence genome

Gene	Chromosome	Protein positions	N402	NW103	Annotation
g720.t1	N402_101	[290]	['K']	['Q']	C6 zinc finger domain protein [*Aspergillus niger* CBS 513.88]
g1650.t1[a]	N402_200	[167]	['S']	['F']	thioredoxin reductase [*Aspergillus niger* CBS 513.88]
g2096.t1	N402_200	[416]	['S']	['F']	hypothetical protein ASPNIDRAFT_36304 [*Aspergillus niger* ATCC 1015]
g2973.t1	N402_301	[570]	['Y']	['S']	RHS Repeat protein [*Aspergillus niger* CBS 513.88]
g3183.t1	N402_301	[458]	['K']	['E']	tyrosyl-DNA phosphodiesterase [*Aspergillus niger* CBS 513.88]
g3203.t1	N402_301	[21]	['Q']	['R']	esterase family protein [*Aspergillus niger* CBS 513.88]
g5753.t1	N402_401	[256]	['Y']	['F']	phosphoketolase [*Aspergillus niger* CBS 513.88]
g7045.t1[b]	N402_501	[26]	['A']	['D']	hypothetical protein ASPNIDRAFT_41124 [*Aspergillus niger* ATCC 1015]
g7360.t1[b]	N402_502	[147, 153]	['T', 'T']	['A', 'A']	hypothetical protein ASPNIDRAFT_41240 [*Aspergillus niger* ATCC 1015]
g7523.t1	N402_603	[26]	['Q']	['R']	hypothetical protein ANI_1_2258184 [*Aspergillus niger* CBS 513.88]
g7928.t1	N402_601	[2244]	['R']	['G']	polyketide synthase [*Aspergillus niger* CBS 513.88]
g8311.t1	N402_601	[261]	['L']	['-']	MFS transporter [*Aspergillus niger* CBS 513.88]
g8370.t1	N402_601	[54]	['G']	['S']	aldehyde dehydrogenase [*Aspergillus niger* CBS 513.88]
g9214.t1	N402_700	[535]	['E']	['D']	ubiquitin-protein ligase Ufd4 [*Aspergillus niger* CBS 513.88]
g9420.t1	N402_700	[447]	['D']	['H']	WD repeat protein [*Aspergillus niger* CBS 513.88]
g10171.t1	N402_800	[96]	['Q']	['E']	BRCT domain protein [*Aspergillus niger*]
g10473.t1	N402_800	[236]	['I']	['L']	pre-mRNA-processing factor 31 [*Aspergillus niger* CBS 513.88]
g10704.t1[b]	N402_800	[760]	['H']	['P']	hypothetical protein ASPNIDRAFT_187743 [*Aspergillus niger* ATCC 1015]

[a] indicates the mutation linked to the *goxB* locus

[b] indicates the mutation found in the other *gox* mutants
